# Hypoglycemic, Antiglycation, and Cytoprotective Properties of a Phenol-Rich Extract From Waste Peel of *Punica granatum* L. var. Dente di Cavallo DC2

**DOI:** 10.3390/molecules24173103

**Published:** 2019-08-27

**Authors:** Antonella Di Sotto, Marcello Locatelli, Alberto Macone, Chiara Toniolo, Stefania Cesa, Simone Carradori, Margherita Eufemi, Gabriela Mazzanti, Silvia Di Giacomo

**Affiliations:** 1 Department of Physiology and Pharmacology “V. Erspamer”, Sapienza University, P.le Aldo Moro 5, 00185 Rome, Italy; 2 Department of Pharmacy, University “G. D’Annunzio” of Chieti-Pescara, Via dei Vestini 31, 66100, Chieti, Italy; 3 Department of Biochemical Sciences “A. Rossi Fanelli”, Sapienza University, P.le A. Moro 5, 00185 Rome, Italy; 4 Department of Environmental Biology, Sapienza University, P.le Aldo Moro 5, 00185 Rome, Italy; 5 Department of Chemistry and Technology of Drugs, Sapienza University, P.le Aldo Moro 5, 00185 Rome, Italy

**Keywords:** vegetable waste, phenolics, hyperglycemia-induced oxidative stress, α-glucosidase, α-amylase, antioxidant activity, inhibition of advanced glycation end-products, chelating activity

## Abstract

Pomegranate peel is a natural source of phenolics, claimed to possess healing properties, among which are antioxidant and antidiabetic. In the present study, an ethyl acetate extract, obtained by Soxhlet from the peel of Dente di Cavallo DC2 pomegranate (PGE) and characterized to contain 4% *w*/*w* of ellagic acid, has been evaluated for its hypoglycemic, antiglycation, and antioxidative cytoprotective properties, in order to provide possible evidence for future nutraceutical applications. The α-amylase and α-glucosidase enzyme inhibition, interference with advanced glycation end-products (AGE) formation, and metal chelating abilities were studied. Moreover, the possible antioxidant cytoprotective properties of PGE under hyperglycemic conditions were assayed. Phenolic profile of the extract was characterized by integrated chromatographic and spectrophotometric methods. PGE resulted able to strongly inhibit the tested enzymes, especially α-glucosidase, and exerted chelating and antiglycation properties. Also, it counteracted the intracellular oxidative stress under hyperglycemic conditions, by reducing the levels of reactive oxygen species and total glutathione. Among the identified phenolics, rutin was the most abundant flavonoid (about 4 % *w*/*w*). Present results suggest PGE to be a possible remedy for hyperglycemia management and encourage further studies to exploit its promising properties.

## 1. Introduction

Fruit and vegetable waste (i.e., seeds, peel, rind, and pomace) are defined as the inedible parts of vegetables, discarded during collection, handling, transportation, and processing [1]. Waste management is an important issue of the modern society, due to its environmental and economic impact. It has been reported that up to 87% of fruits, vegetables, and cereals are discarded before reaching consumers, thus posing disposal and environmental problems [2]. Nevertheless, waste could be reduced by applying targeted prevention strategies, among which are reduction, reuse, and recycling [2]. Particularly, recycling strategies (e.g., composting, processing to flour, conversion into water) allow to recover waste after major modifications and to reuse them for future applications or as starting material for the extraction of specific compounds [3,4]. 

In support, several crop waste, from both vegetable, cereals and fruits, are known to be source of bioactive phytochemicals, among which are polyphenols, anthocyanins, carotenoids, terpenoids, lactones, glucosinolates, lectins and dietary fibers [4,5,6,7,8,9,10,11] and to possess interesting pharmacological activities [5,6,7,8,9,10,11,12,13,14,15,16], thus becoming attractive materials for further uses in drug discovery and nutraceutical fields.

Among phytochemicals, polyphenols represent a large class of secondary metabolites with diverse chemical structures and functions, usually concentrated in the rind, peel, and seeds of fruits and vegetables [5]. Often, these compounds have been found to be more concentrated in the peel and other waste of different fruits (e.g., banana, apples, peaches, and pears) and vegetables (e.g. eggplant, pepper, and potato) with respect to the edible portions [7,17,18], and responsible in several biological activities [6,7,8,18,19,20,21]. 

For instance, phenolic enriched extracts from stigmas and tepals plus anthers of saffron exerted protective properties in *in vitro* and *ex vivo* models of inflammation and oxidative stress [19], whereas a polyphenol rich extract from the peel of DR2 eggplant produced antioxidant and antiviral effects towards herpes simplex virus type 1 [7]. Similarly, the extracts from root of orchid were found to contain high phenolic amount, and to possess interesting antioxidant activities [20]. At last, the ethanolic extracts from the peel and seeds of red pepper displayed both antioxidant and antimutagenic properties and inhibited glycolytic enzymes, likely ascribable to the presence of phenolic compounds [21].

Pomegranate (*Punica granatum* L.) peel is known to be an attractive source of phenolic compounds, with an amount about 10-fold higher than that of the pulp [22]. All of the parts of the pomegranate plant, including the edible part, juice, and the nonedible peel, seeds, and flowers have been widely assessed for possible healing effects, thus highlighting antimicrobial, antioxidant, antiinflammatory, antiproliferative, hypolipidemic, and hypoglicemic properties [8,23,24,25,26,27,28]. Particularly, peel byproducts have been approached as possible natural additives for food preservation and quality enhancement, as well as components of food supplements and nutraceuticals, in order to exploit their health-promoting features [23]. 

The potential healing effects of pomegranate byproducts are mainly ascribed to the polyphenolic compounds, among which are ellagitannins (i.e., punicalagins, punicalin, and gallagic acid), phenolic acids (i.e., gallic acid and ellagic acid), anthocyanins, and flavonoids, whose composition depends not only on varieties, plant parts, and environmental factors, but also on the extraction methods [23].

In line with this knowledge, the present study aimed at evaluating the possible healing properties of an ethyl acetate extract (PGE), obtained by Soxhlet apparatus, from the peel fruit of Dente di Cavallo DC2 pomegranate. This represents one of the most important Italian cultivars, widely cultivated for both ornamental and food purposes, due to its ability to grow under adverse environmental conditions, particularly drought and cold, and to produce a high-yield, sweet-flavored, and intense red color fruit juice [29]. Therefore, providing possible evidence for future applications of the peel fruit from Dente di Cavallo DC2 pomegranate can represent an interesting recycling strategy of vegetable waste as valuable starting material for cosmetic or nutraceutical industry, while reducing cost of waste disposal and management for pomegranate juice industry. 

PGE has been previously characterized to be rich of phenolics, with a 5.4:1 *w*/*w* ratio between ellagic acid and punicalagins, and a very low anthocyanin amount (about 0.002% *w*/*w*) [30]. Considering that phenolic acids, among which are ellagic, gallic and ferulic acids, are known to regulate carbohydrate metabolism and hepatic glucose homeostasis through different mechanisms [31,32], in the present study, the potential *in vitro* hypoglycemic and antiglycation properties of PGE, in terms of inhibition of α-amylase and α-glucosidase, key enzymes responsible for dietary carbohydrates digestion, along with its metal chelating ability and interference with the advanced glycation end-products (AGE) formation, were evaluated. Moreover, considering the previously reported antioxidant and radical scavenging properties of PGE [30], its possible cytoprotective effects towards the oxidative stress associated with hyperglycemia have been evaluated. 

The phenolic profile of PGE was further characterized by integrated high-performance thin-layer (HPTLC) and high-performance liquid with photodiode array detection (HPLC-PDA) chromatographic techniques and spectrophotometric assays, in order to identify the possible bioactive phytochemicals. To the best of our knowledge, this is the first study focused on the peel from Dente di Cavallo DC2 pomegranate as a possible source of bioactive phytocomplex for further nutraceutical applications.

## 2. Results and Discussion

### 2.1. Phytochemical Analysis

Spectrophotometric analysis showed that PGE contained high levels of total phenolics and tannins, expressed as microgram equivalents of tannic acid (TAE) per milligram of extract (Table 1): These data are in agreement with the previous characterization that highlighted PGE to contain the greatest amount of phenolics with respect to the ethanolic and methanolic pomegranate peel extracts [30]. Considering that we found a phenolics/tannins ratio of 3.9 *w*/*w*, tannins can be estimated to be about 40% of total phenolics. Also, our results highlighted that PGE contained about a 20% *w*/*w* of total flavonoids (expressed as quercetin equivalents). Taking into account the overall yield obtained for PGE (about 1.09% *w*/*w*) [30], a higher than 2 g/kg total flavonoid amount in the fresh material can be estimated: On the basis of the Peterson and Dwyer classification [7], the peel fruit of Dente di Cavallo DC2 pomegranate results to contain high flavonoid levels, thus confirming its possible interest as a nutraceutical source. Nevertheless, the true bioavailability of these phytochemicals after ingestion remains to be evaluated.

**Figure 1:**
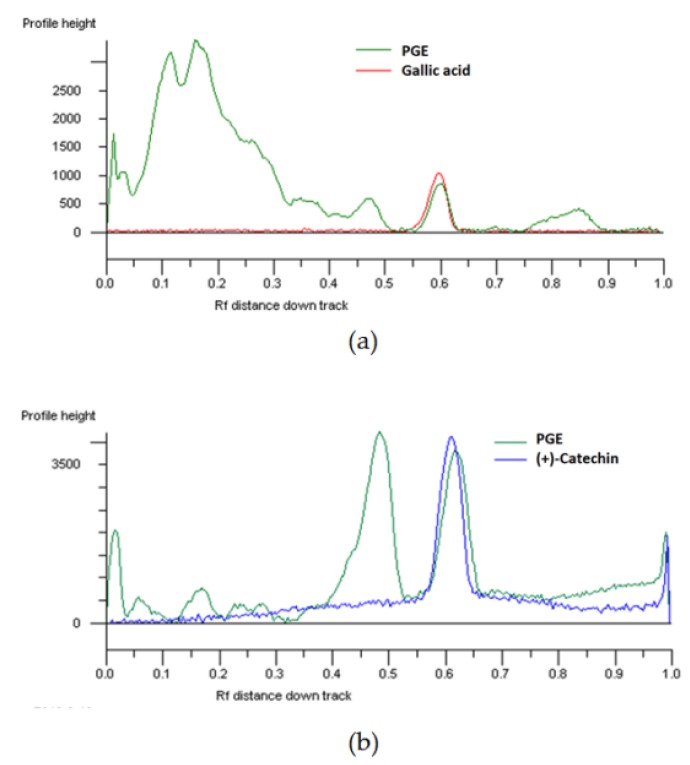
HPTLC chromatogram of the ethyl acetate extract, obtained by Soxhlet from the waste peel of *Punica granatum* L. var. Dente di Cavallo DC2 (PGE), and the standard gallic acid (**a**; Rf 0.58–; visualization under UV 366 nm after NPR derivatization) and (+)-catechin (**b**; Rf 0.58–UV 366 nm after derivatization with NPR and anisaldehyde).

The presence of different phenolics in PGE was also highlighted by HPTLC analysis and visualized by means of suitable derivatization reagents (Figure S1): among them, gallic acid and catechin were identified (Figure 1).

Phenolic profile was also detected and quantified by HPLC-PDA analysis, whose results agreed with the integrated analysis described above (Table 2). Particularly, PGE resulted to contain a high amount of rutin (about 4% *w*/*w*), along with catechin (about 0.27% *w*/*w*), 2,3-dimethoxy benzoic acid (about 0.2% *w*/*w*), gallic acid (about 0.1% *w*/*w*), and syringic acid (about 0.06% *w*/*w*). As previously reported [30], PGE also contained about a 4% *w*/*w* of ellagic acid and 0.7% of total punicalagins (i.e., anomers α and β), with low levels of anthocyanins (0.002% *w*/*w*). This phytochemical profile highlighted a 1:1 ratio between the most abundant phenolics, ellagic acid and rutin. Conversely, other common phenolics, such as chlorogenic acid, *t*-cinnamic acid, *o*- and *p*-coumaric acid, *t*-ferulic acid, and quercetin, were not found.

Published literature highlights that peel of pomegranate is characterized by a typical pool of phenolics (predominantly those from hydrolysable tannins), with punicalagin and its metabolites as the major and most studied compounds. Particularly, the amount of hydrolyzable tannins has been reported to be in the range of 27–172 g/kg, with a prevalence of monomeric phenolics [33]. Tannins are mainly characterized by gallic acid and ellagic acid esters (i.e., gallotannins and ellagitannins), including the gallagylesters punicalin and punicalagin, with lower amounts of hydroxybenzoic acids [33]. 

Li et al. identified catechin, epicatechin, and low levels of rutin, chlorogenic, and caffeic acid, along with punicalagin, and ellagic and gallic acids, in the peel from Chinese pomegranate [34]. Accordingly, gallic acid, ellagic acid, caffeic acid, *p*-coumaric acid, quercetin, and vanillic acid were found to be the predominant compounds in the peel from Tunisian varieties of pomegranate [35]. Phenolic composition of the extracts from pomegranate peel can be varied also as a consequence of extraction method. 

They are usually extracted using methanol, ethanol, hydroalcoholic solutions [36,37], or water under elevated temperature and extended time, with possible consequent oxidation of certain phenolics [38]. Also, applying specific methodologies, such as sonication, supercritical CO_2_, increasing extraction temperatures, microwave, or ultrasound-assisted extraction, can improve the yield of total phenolics [38,39,40,41,42]. Wang et al. reported that methanol, ethanol, acetone, and water generally yielded a significant co-extraction of phenolics, proanthocyanidins, and flavonoids, while decreased the yield of target antioxidants; nevertheless, ethyl acetate seems to favor the selective extraction of antioxidants [38].

In this context, extraction by ethyl acetate in Soxhlet of the peel fruit from Dente di Cavallo DC2 pomegranate allowed to obtain high levels of ellagic acid, similarly to maceration in ethyl acetate, despite a significant lowering of punicalagins (about 0.2:1 ratio between punicalagins and ellagic acid) [30]. Conversely, the ethanolic extract was most abundant in punicalagins, with a lower amount of ellagic acid (about a 14:1 ratio between punicalagins and ellagic acid). Accordingly, partitioning between water and ethyl acetate has been reported to increase ellagic acid content, along with the antioxidant potency of the pomegranate peel extracts [43]. This confirms that extraction in ethyl acetate by Soxhlet could represent a suitable method for selection of bioactive phenolic acids and flavonoids from pomegranate peel.

### 2.2. Metabolic Enzyme Inhibition

Multiple evidences have highlighted that dietary phenolics can be responsible for antidiabetic effects, likely due to the ability of these compounds to regulate the key pathway of carbohydrate metabolism [31]. Some of them have been reported to decrease the activity of α-amylase and α-glucosidase, two important enzymes responsible for digestion of dietary carbohydrates to glucose [44]. Particularly, salivary and pancreatic α-amylases catalyze the endo-hydrolysis of α-1,4-glucosidic linkages of amylose, while α-glucosidases are responsible for further digestion of terminal α-1,4-glucosidic linkages in the small intestinal brush border, allowing glucose release and absorption across the intestinal enterocytes via specific transporters [44]. Therefore, inhibiting these enzymes can allow to reduce the rate of glucose release in the small intestine and to suppress postprandial hyperglycemia and formation of glycated end-products, with further complications. 

In line with this evidence and taking into account the peculiar phenolic composition of PGE, the ability of the extract to affect the function of both enzymes as possible mechanisms for glycemia control has been studied.

**Figure 2:**
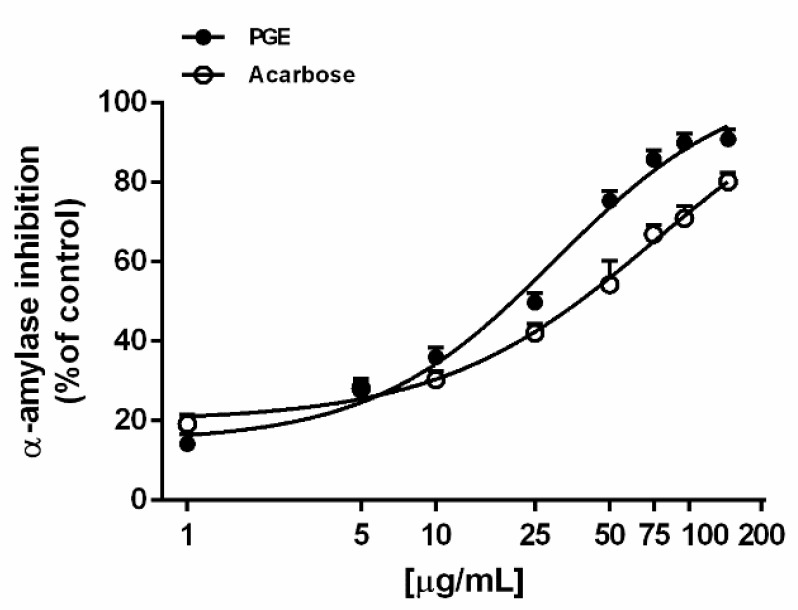
α-Amylase inhibition by the ethyl acetate extract, obtained by Soxhlet from the waste peel of *Punica granatum* L. var. Dente di Cavallo DC2 (PGE), with respect to the reference standard acarbose. Each value represents mean ± SEM (*n* = 4).

Under our experimental conditions, PGE was found able to inhibit the α-amylase enzyme in a concentration-dependent manner, being effective already at lower concentrations, similarly to the positive control acarbose (Figure 2). On the basis of the IC_50_ values, the potency of PGE resulted about 2.5-fold higher than that of acarbose (Table 3).

**Figure 3:**
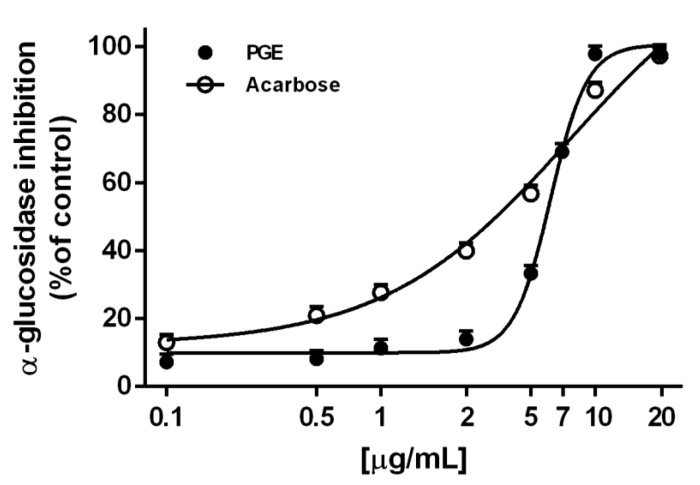
α-Glucosidase inhibition by the ethyl acetate extract, obtained by Soxhlet from the waste peel of *Punica granatum* L. var. Dente di Cavallo DC2 (PGE), with respect to the reference standards acarbose. Each value represents mean ± SEM (*n* = 4).

Also, PGE produced a marked and concentration-dependent α-glucosidase inhibition, achieving the maximum effect at 10 µg/mL (Figure 3). The effect was similar to that produced by the positive control acarbose, as confirmed by the IC_50_ values (Table 3). Conversely, the extract resulted ineffective towards lipase enzyme, thus suggesting its selective activity for enzymes involved in carbohydrate metabolism (Table 3).

On the basis of the Pearson correlation analysis, the inhibition of these enzymes by PGE appeared not to be significantly correlated, being the extract about 5-fold more potent in inhibiting α-glucosidase (Table 4). This suggests that PGE is able to affect the activity of both enzymes, although different compounds or inhibitory mechanisms can be responsible for the higher potency as an α-glucosidase inhibitor.

Based on the evidence that several flavonoids are able to inhibit the enzymes involved in the carbohydrate metabolism, although with different specificity and potency [45,46], and considering that PGE exerted the highest potency as an α-glucosidase inhibitor, we also assessed the possible contribution of rutin to PGE inhibition, being the most abundantly identified flavonoid. Under our experimental conditions, rutin significantly inhibited α-glucosidase enzyme, with a potency similar to those of acarbose and PGE, as confirmed by the IC_50_ values (Table 3). Our results agree with the literature, which highlights rutin to possess glycolytic enzyme inhibitory and antiglycation properties [47,48]. It also exhibited an acarbose-like and specific inhibitory effect on maltase activity in the intestine, and reduced glycemia when administered before glucose overload [49]. Rutin has been reported to deactivate α-amylase and α-glucosidase by forming inactive complex [50] and to be more effective than the derived flavonol quercetin [46].

Among the other identified compounds of PGE, gallic acid has been reported to affect both α-amylase and α-glucosidase [51], and to be useful to reduce side effects of acarbose when used in combination [52]. Conversely, Kam et al. highlighted that gallic and ellagic acids were effective towards α-glucosidase, while producing only a weak inhibition of α-amylase [53]. *In silico* studies have also highlighted catechin and syringic acid to possess inhibitory properties towards α-glucosidase and α-amylase, respectively [46]. In regard to the minor components of PGE, the contribution of punicalagins seems to be of lower relevance, taking into account that previous data highlighted the ethanolic extract from the peel of Dente di Cavallo DC2 pomegranate, characterized by a high amount of punicalagins with respect to ellagic acid (14:1 ratio) [30], was ineffective as an α-glucosidase inhibitor (data not shown). Altogether, this evidence supports our hypothesis about the possible contribution of rutin to the PGE activity. Taken together, these results suggest that PGE may contain a pool of phenolics able to differently affect several targets involved in the control of carbohydrate metabolism: Particularly, the high potency against glucosidase seems to be ascribable to the combined contributions of rutin, catechin, ellagic acid, and gallic acid, while rutin and syringic acid could contribute to the amylase inhibition. Nevertheless, tangled interactions among compounds in the PGE phytocomplex cannot be excluded. 

Furthermore, the high potency of PGE as an α-glucosidase inhibitor is noteworthy and promising, considering that α-glucosidase inhibitor drugs are reported to be useful for adequate control of type 2 diabetes, despite the side effects and costs that limit their market [54]. In this context, PGE could represent a suitable natural alternative to the available treatments for the management and prevention of diabetes and associated ailments to be further studied and developed.

### 2.3. Iron Chelating Activity

**Figure 4:**
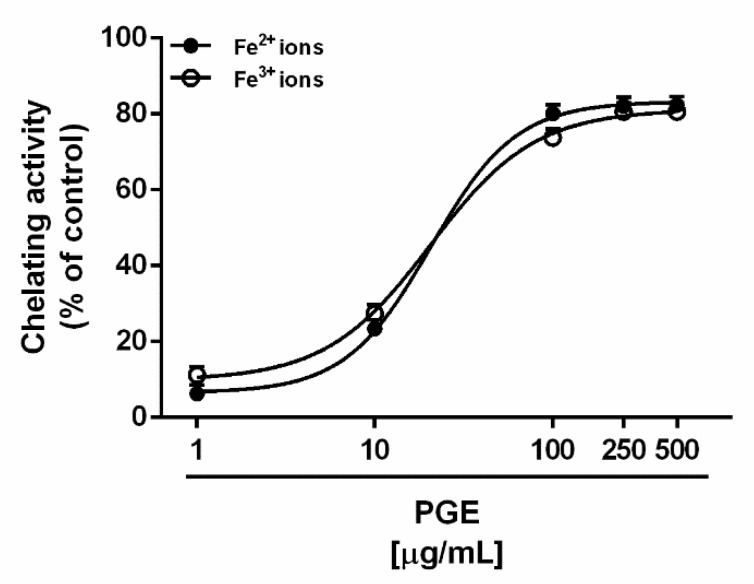
Chelating activity exhibited by the ethyl acetate Soxhlet extract of waste peel of *Punica granatum* L. var. Dente di Cavallo DC2 (PGE) towards ferrous (Fe^2+^) and ferric (Fe^3+^) ions. Each value represents mean ± SEM (*n* = 4).

Alterations of iron and copper homeostasis are characteristic features of diabetes, evidenced by deposition of iron and copper in different tissues and increased urinary excretion. Therefore, chelating therapies have been suggested as possible strategies to counteract metal-catalyzed oxidation reactions and the production of reactive oxygen species (ROS), through blocking the Fenton cascade, thus preventing the development of hyperglycemia complications [55]. In the present study, PGE exhibited similar ferrous and ferric ion chelating activities, achieving about the maximum effects of 80% at the concentration of 100 μg/mL (Figure 4); accordingly, the IC_50_ values were not statistically different (Table 4).

According to the results of Pearson analysis, the chelating abilities of PGE towards ferrous and ferric ions appear to be correlated each other, although they were not correlated with the glycolytic enzyme inhibition (Table 4). Also, as expected, a significant correlation was found with the inhibition of AGE formation (Table 4). Interestingly, the extract was about 20- and 3-fold more potent than the standard flavonoids rutin and quercetin, respectively. This suggests that the PGE phytocomplex can contain more potent bioactive compounds, or that possible synergistic or additional effects among its constituents allow to improve the activity, with respect to the pure compounds.

Among the identified compounds, ellagic acid was reported to be able to form complexes with several metal ions and to produce antioxidative effects through reducing the iron-mediated free radical formation by chelating mechanisms [56]. Also, the free radical scavenging effects of rutin and the inhibition of lipid peroxidation were ascribed to its ability to chelate iron ions, thus leading to the formation of metal-complexes that slightly promote free radical reactions [57,58]. Interestingly, catechin was found to exert cytoprotective and antiradical activities, likely through its iron-chelating abilities [59,60]. Finally, for gallic and syringic acid, a lower iron-binding efficiency, likely due to the chemical features, was reported [61,62].

On the basis of this evidence, different phenolics—among which ellagic acid, rutin, and catechin—seem to likely contribute to the iron chelating abilities of PGE.

### 2.4. Advanced Glycation End-Product (AGE) Inhibition

The formation of advanced glycation end-products (AGE) results from a non-enzymatic reaction between the carbonyl group of reducing sugars and a free amino group of proteins, to form reversible Schiff’s base, which undergoes rearrangement to Amadori products, which in turn undergo a series of modifications to form stable, heterogeneous adducts [63]. Moreover, a variety of other pathways such as autoxidation of glucose, or ascorbate and lipid peroxidation, can lead to AGE formation [32]. AGE formation deserves special attention due to its involvement in the development and progression of different pathologies (such as atherosclerosis, nephropathies, and retinopathy), particularly diabetes complications [63,64]. They are considered to be glycotoxins, due to their ability to increase oxidant stress and inflammation [65].

Among the proposed mechanisms for AGE damage, they seem to activate specific cellular receptors, namely RAGE (receptor for advanced glycation endproducts), which have been found highly expressed during pathogenic and stress conditions, thus inducing the gene expression of cell-specific pro-inflammatory and oxidative signaling pathways [66]. Also, under oxidative stress, tissue accumulation of AGE has been reported to cause upregulation of the matrix metalloproteinases, which are associated with chronic inflammatory conditions [65]. Therefore, inhibiting AGE formation at different steps of the glycation process [65] has been highlighted to represent a possible therapeutic target for the prevention and treatment of different chronic pathologies, particularly diabetes complications.

**Figure 5:**
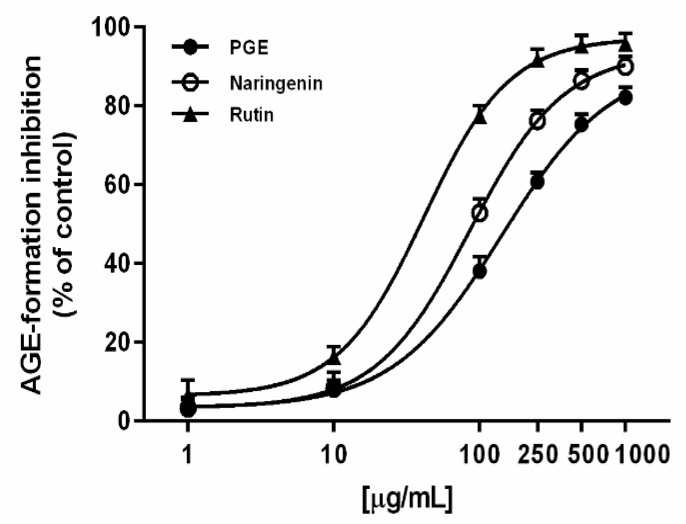
AGE inhibition by the ethyl acetate extract, obtained by Soxhlet from the waste peel of *Punica granatum* L. var. Dente di Cavallo DC2 (PGE), rutin, and the positive control naringenin. Each value represents mean ± SEM (*n* = 4).

Under our experimental conditions, PGE produced a marked and concentration-dependent inhibition of AGE, achieving the maximum effect at 1000 µg/mL (Figure 5). However, its potency was about 1.7-fold lower than the positive control naringenin, as confirmed by the IC_50_ value (Table 3). Rutin, the most abundant flavonoid identified in PGE, produced a concentration-dependent inhibition of the AGE levels (Figure 5), with about a 3-fold greater potency than PGE (Table 3); it was also effective as an AGE inhibitor at the concentration found in PGE, thus suggesting a possible contribution to the activity of the extract. The Pearson correlation analysis highlighted that AGE inhibition by PGE was not significantly correlated with inhibition of glycolytic enzyme, despite a significant correlation with iron chelating activity (Table 4). Similarly, AGE inhibition by rutin was not significantly correlated with its glycolytic enzyme inhibition. This behavior supports our hypothesis about the contribution of rutin to PGE bioactivity, although the involvement of all the phytocomplex appears to be likely.

Our results agree with previous published literature, which highlights rutin and its derived flavonol as possessing antiglycation *in vivo* properties, being able to prevent AGE formation through free radical scavenging and metal chelating mechanisms [67]. Particularly, structure–activity relationship (SAR) studies have highlighted that the presence of 3,4-dihydroxyl groups in the B-ring of flavonoid is required for the antiglycation effects of rutin through free radical scavenging activity [65]. Moreover, gallic acid and catechin have been reported to prevent AGE formation by trapping α-dicarbonyl compounds or inhibiting Amadori product formation [68,69].

The antiglycation activity of some polyphenols—among which gallic acid—seems to be mediated by the upregulation of peroxisome proliferator-activated receptors (PPAR), which play a pivotal role in the control of carbohydrate and lipid metabolisms and in the downregulation of RAGE expression [32]. Particularly, a pomegranate flower extract rich in gallic acid has been shown to increase PPAR-gamma mRNA expression, along with enhancing sensitivity to the insulin receptor, thus suggesting that this mechanism could be involved in the control of AGE formation [70]. Similarly, ellagic acid has exhibited interesting antiglycation properties, both *in vitro* and *in vivo*, by reducing the expression of RAGE along with that of specific regulatory factors for angiogenesis and hypoxia, thus suggesting it can represent a potent antiglycating agent to be further developed [71]. This evidence, in agreement with the previously described antioxidant, glycolytic enzyme inhibitory, and iron chelating properties, strengthens our hypothesis about the possible role of PGE phytocomplex in the control of hyperglycemia-related ailments, and suggests the need of further *in vivo* evaluation of its potential usefulness in diabetic animal models.

### 2.5. PGE Counteracts the Oxidative Stress Induced Under Hyperglycemia

Recent evidence has suggested that diabetes can be considered as an oxidative stress disorder, occurring as a consequence of an imbalance between free radical formation and reduced ability of the physiological antioxidant defences [72]. Oxidative stress also occurs as a consequence of mitochondrial dysfunction and alternative metabolism of glucose (mainly polyol pathway) under hyperglycaemic conditions, and in turn, contributes to diabetes complications [73]. Therefore, free radicals can oxidize cell structures, including protein, lipid, and nucleic acid, and lead to AGE and ALE (advanced lipoxidation end-product) formation. AGE accumulation can further contribute to increased oxidative stress and inflammation in the tissues, through the activation of the AGE–RAGE axis and specific pro-oxidant and pro-inflammatory pathways [32]. Therefore, counteracting oxidative stress during hyperglycemia conditions and diabetes appears to be a suitable strategy to limit the oxidative cascade damage and complication development. In this context, and considering the previously described activities of PGE, we also studied the antioxidant cytoprotective ability of our extract under hyperglycemia in a gastric cancer cell model, known to be characterized by a glycolytic metabolism [74] and increased oxidative stress in the presence of high-glucose concentrations [75].

Preliminary cytotoxicity assays showed that PGE did not significantly affect cell viability of human gastric carcinoma AGS cells up to the concentration of 100 μg/mL and 72 h exposure (data not shown). On the basis of this evidence, this concentration was chosen as the highest to be assessed in the further assays under hyperglycemia. Also, the glucose concentrations of 3.0, 4.5, 6.0, and 7.5 mg/mL were found to progressively increase the intracellular ROS levels with respect to basal oxidation levels obtained at a final glucose concentration of 1.4 mg/mL, without affecting cell viability. Particularly, after 72 h incubation, ROS levels increased up from 3.7- to 5.3-fold within the range of the tested concentrations (data not shown). The glucose concentration of 6.0 mg/mL, at which a submaximal 5-fold ROS increase with respect to the basal level was observed, was chosen for further cytoprotective assays in the presence of nontoxic concentrations of PGE (i.e., 10, 50, and 100 μg/mL). These data agree with previous published studies, in which 7.8 mM glucose (corresponding to about 1.4 mg/mL) [76] was used as a non-fasting basal blood sugar level, while concentrations of 17.5, 25, and 33 mM glucose (corresponding to about 3.0, 4.5, and 6.0 mg/mL) were employed to simulate hyperglycemic conditions [76,77,78].

**Figure 6:**
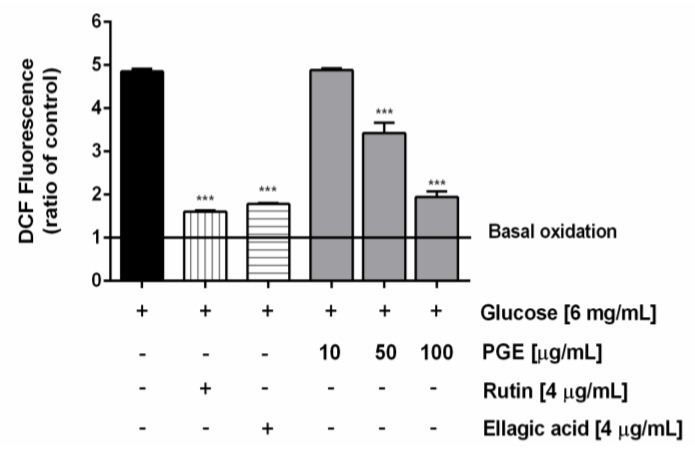
Effect of the ethyl acetate extract, obtained by Soxhlet from the waste peel of *Punica granatum* L. var. Dente di Cavallo DC2 (PGE), rutin, and ellagic acid on the reactive oxygen species (ROS) levels induced by hyperglycemia conditions in AGS cells. ROS levels are expressed as oxidation index with respect to the basal levels. Data are mean ± SE from at least two independent biological replicates (*n* = 2). *** *p* < 0.001 vs Glucose [6 mg/mL] by ANOVA followed by Dunnett’s Multiple Comparison Post Test.

**Figure 7:**
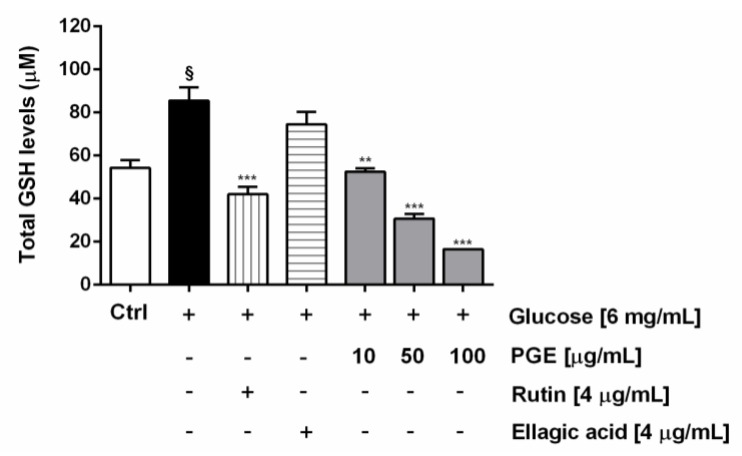
Effect of PGE, rutin, and ellagic acid on total GSH levels induced by hyperglycemia conditions in AGS cells. Total GSH was evaluated in cell lysates and calculated with respect to the calibration curves of GSH and GSSG. Data are mean ± SE from at least two independent biological replicates (*n* = 2). ^§^ *p* < 0.01 vs control by Student *t*-test; ** *p* < 0.01 and *** *p* < 0.001 vs Glucose [6 mg/mL] by ANOVA followed by Dunnett’s Multiple Comparison Post Test.

Along with the extract, the most abundant identified phenolics, i.e., ellagic acid and rutin, were assayed at the concentration of 4 μg/mL, corresponding to the level detected at the highest nontoxic concentration of PGE (100 μg/mL). Under our experimental conditions, PGE produced a statistically significant and concentration-dependent reduction of the ROS levels induced by hyperglycemia (glucose concentration of 6 mg/mL), achieving a maximum lowering of about 60% at the highest tested concentration. Likewise, both rutin and ellagic acid produced a similar (63 and 67%, respectively) lowering of hyperglycemia-induced ROS levels (Figure 6). Also, total glutathione (GSH) levels were found significantly raised by hyperglycemia, achieving about a 33% increase with respect to the basal levels. The treatment with PGE significantly reduced the total GSH levels, achieving a reduction from about 50% to a maximum 80% within the tested concentrations (Figure 7). Similarly, rutin produced a 41% lowering of GSH, despite a lacking effect of ellagic acid at the concentration found in 100 μg/mL of PGE (Figure 7).

ROS are physiologically produced under aerobic metabolism and their production may be increased during cell injury, like hyperglycemia. Considering that excessive ROS levels may lead to cell damage and death, in order to prevent irreversible damage and to restore the redox homeostasis, an adaptive cell response by upregulation of antioxidant systems occurs. Among them, GSH plays a pivotal role in maintaining redox homeostasis, and high levels have been found in various tumors, likely as a mechanism of cancer cytoprotection and resistance [79]. It is known that antioxidants may directly inhibit ROS by scavenging and inactivating mechanisms, or indirectly by inhibiting their formation, through metal chelation, or by affecting physiological defences, including reduced glutathione, superoxide dismutase, catalase, and glutathione reductase [80]. Accordingly, under our experimental conditions, GSH and ROS levels were strongly increased by the hyperglycemic oxidative stress in AGS cells, while PGE was able to deplete GSH, likely as a consequence of the reduced ROS levels. Previous evidence has highlighted that PGE can act by both radical scavenging mechanisms [30], and through metal chelating properties. Also, under hyperglycemia, it resulted able to affect further mechanisms of ROS generation, strictly connected with carbohydrate metabolism and diabetes, by inhibiting glycolytic enzymes and AGE formation. Altogether, this evidence supports our hypothesis about the ability of PGE to counteract the oxidative stress occurring during hyperglycemia, likely acting through direct and indirect antioxidant mechanisms, thus lowering the need of antioxidant cell defences. Interestingly, both phenolics rutin and ellagic acid were able to affect the ROS levels induced by hyperglycemia in AGS cells, while only rutin was effective in depleting GSH levels at the concentration found in the extract. This behavior strengthens our hypothesis about the involvement of different phenolics in PGE bioactivities, and suggests that using the PGE phytocomplex, instead of pure phenolics, may be a suitable strategy to exploit numerous healing properties, along with possible synergistic interactions occurring among phytochemicals.

## 3. Materials and Methods

### 3.1. Chemicals

All the chemicals, if not otherwise specified, and the RPMI 1640 medium were obtained from Sigma-Aldrich Co (St. Louis, MO, USA). Sodium carbonate, Folin-Ciocalteu’s phenol reagent, tannic acid, aluminium chloride hexahydrate, and the analytical grade solvents ethyl acetate and n-butanol were purchased from Merck (Darmstadt, Germany). Standard flavonoids and phenylpropanoids (>95% purity) were obtained from synthesis and checked by nuclear magnetic resonance spectroscopy. Fetal bovine serum was obtained from Gibco, while the other reagents for antiviral studies, if not otherwise specified, were purchased from Invitrogen (Carlsbad, CA, USA). Anisaldehyde was prepared as a 0.5% *v*/*v* solution in an ice-cooled mixture of sulphuric acid, methanol, and acetic acid (1:17:2 *v*/*v*/*v*). Natural Product Reagent (NPR) was obtained by preparing a 0.5% *w*/*v* solution of diphenylborinic acid aminoethylester in ethyl acetate.

### 3.2. Plant Material and Extract Preparation

Fruits of Italian *Punica granatum* L. Dente di Cavallo DC2 cultivar were kindly provided at eating ripeness by the local Italian farm Giovomel (Avellino, Italy). To prepare the PGE extract, peel (exocarp and mesocarp) of pomegranate fruit was separated from arils, then washed, triturated in a blender for 30 s, and subjected to Soxhlet extraction in ethyl acetate, as reported by Masci et al. [30]. The obtained extract was dried at 40 °C in the dark, stored under ultrafrost conditions (–18 °C) and dissolved in ethanol or methanol (100% *v*/*v*) for phytochemical and biological analysis.

PGE was previously characterized to be rich in phenolic compounds, particularly ellagic acid (3.77% *w*/*w*) and punicalagins (0.68% *w*/*w*), with a 5.6:1 *w*/*w* ratio, and low levels of anthocyanins (about 0.002% *w*/*w*) [30].

### 3.3. Phytochemical Analysis

#### 3.3.1. Determination of Total Polyphenol, Tannins, and Flavonoid Content

The total amounts of both phenolics and tannins were measured by the spectrophotometric Folin–Ciocalteu method and calculated as tannic acid equivalents (TAEs), while those of flavonoids were determined by the aluminium trichloride method and expressed as quercetin equivalents (QE), according to previous published methods [7].

#### 3.3.2. Chromatographic Analysis of Phenolics

Phenolic compounds of PGE were analyzed by integrated high-performance thin-layer chromatography (HPTLC) and high-performance liquid chromatography (HPLC), according to the methods reported by Di Sotto et al. [7]. For the HPTLC, a methanolic solution of PGE (50 mg/mL) was analyzed in comparison with standard phenolics (1 mg/mL), among which were phenolic acids (i.e., chlorogenic acid, caffeic acid, and gallic acid), flavonoids (i.e., rutin, quercetin, and kaempferol), and catechin. Compounds were identified by comparison with selected standards (Rf values, colors, UV spectra) and literature data. Repeatability was determined by running a minimum of three analyses. For measuring the HPLC-PDA phenolic pattern, PGE was dissolved in the mobile phase at a concentration of 1 mg/mL, then directly injected (20 µL) into HPLC-PDA system (HPLC Waters liquid chromatography—model 600 solvent pump, 2996 PDA). According to a previous standardized method [7], rutin, naringin, quercetin, harpagoside, naringenin, carvacrol, gallic acid, chlorogenic acid, syringic acid, benzoic acid, *p*-hydroxy benzoic acid, 2,3-dimethoxy benzoic acid, 3-hydroxy benzoic acid, 3-hydroxy-4-methoxy benzaldehyde, *t*-cinnamic acid, *o*-coumaric acid, *p*-coumaric acid, vanillic acid, sinapinic acid, *t*-ferulic acid, catechin, and epicatechin were used as standard phenolics.

### 3.4. In Vitro Metabolic Enzyme Inhibition

The ability of PGE to inhibit in vitro the α-amylase, lipase, and α-glucosidase enzymes was measured by spectrophotometric assays by using a microplate reader (Epoch Microplate Spectrophotometer, BioTeK). Acarbose and orlistat were included in all the experiments as standard inhibitors (100% enzyme inhibition) for α-amylase, α-glucosidase, and lipase, respectively, while the vehicle represented the maximum enzyme activity. Additional treatments, in which enzyme solution was replaced by buffer solution, were included in order to evaluate the interfering absorbance produced by the extract. The experiments were performed at least in triplicate, and in each experiment, about six replicates were prepared. Data obtained from at least two experiments were pooled in the statistical analysis. The inhibitory activity was calculated as percentage of inhibition with respect to the vehicle control.

#### 3.4.1. α-Amylase Inhibition

The α-amylase activity was determined by the method of dinitrosalicilic acid (DNSA), according to Di Sotto et al. [21], with minor changes. To perform the assay, serial dilutions of the extract (250 μL; 1–100 μg/mL final concentration, 1:2 to 1:5 dilution factor) were pre-incubated with α-amylase (250 μL; 0.5 mg/mL in phosphate buffer solution, corresponding to 25 U/mL) for 10 min at 37 °C. Then, the mixtures were added to with potato starch solution (0.5 % *w*/*v* in acetate buffer 0.1 M, pH 4.5; 250 μL) and incubated for 10 min at 37 °C. The reaction was stopped by adding 500 μL of DNSA reagent (25 mL of 96 mM DNSA solution in water, 8 mL of 5.3 M sodium potassium tartrate solution in 2 M sodium hydroxide and 12 mL of water), which is able to bind reducing sugar, thus inducing the turning of the solution to red, after incubation in a water bath at 100 °C for 5 min. The absorbance was measured at 540 nm.

#### 3.4.2. α-Glucosidase Inhibition

The α-glucosidase activity was measured through the enzymatic hydrolysis of p-nitrophenyl-α-D-glucopiranoside (PNGP) to p-nitrophenol and D-glucose, according to a previous standardized method [21]. To perform the assay, serial dilutions of PGE (25 μL; 0.5–10 μg/mL final concentration, 1:1.1 to 1:2.5 dilution factor) were pre-incubated for 30 min at 37 °C with 50 μL of α-glucosidase (1 U/mL in PBS 0.1 M), and 100 μL of PBS. Then, 25 μL of PNGP (5 mM in PBS) were added to each tube and the solution was vortexed for 5 min. For each treatment, the *p*-nitrophenol presence was determined by the turning of the solution to orange, and its absorbance was determined at 405 nm.

#### 3.4.3. Lipase Inhibition

Lipase activity was measured through the hydrolysis of p-nitrophenyl palmitate (PNP) to *p*-nitrophenol, according to Vitalone et al. [81]. Serial dilutions of the samples (16 μL) were mixed with a lipase solution (5.0 mg/mL in water; 12 μL) and TRIS HCl (75 mM, pH 8.5; 162 μL). The mixture was supplemented with PNP (10 mM; 25 μL) and pre-incubated on ice for 5 min. For each treatment, the absorbance was determined at 405 nm.

### 3.5. Iron Chelating Activity

The activities were evaluated by the ferrozine assay, according to previous published methods [82]. Experiments were performed in acetate buffer (0.1 M, pH 4.5) to optimize the conditions for ferric ion reduction and chelation [82]. Chelation ability was evaluated against both ferrous and ferric ions. In the first case, a freshly prepared solution of FeSO4×7H_2_O (50 µL; 200 µM) in acetate buffer, was mixed for 2 min with the samples (50 µL), and afterwards with acetate buffer (50 µL). Conversely, ferric ion chelating was assessed by mixing a solution of FeCl_3_×6H_2_O (50 µL; 200 µM) in acetate buffer for 2 min with the samples (50 µL), and afterwards with hydroxylamine (50 µL; 5 mM). After the addition of ferrozine solution (50 µL; 5 mM), the absorbance of the complex formed with ferrous ions was measured at 562 nm. The percentage of chelating activity was calculated as follows: 100 (A_control_ − A_sample_)/A_control_, where A_control_ is the absorbance of the vehicle, whereas A_sample_ is that of the tested sample. For each sample, the iron-chelating power was calculated in relation to the positive controls rutin and quercetin.

### 3.6. Advanced Glycation End-Product (AGE) Inhibition

The inhibition of AGE formation by PGE and the standard phenolics naringenin, rutin, and ellagic acid was measured through the method described by Lee et al. [83], with slight modifications. Progressive dilutions of the samples (1–1000 μg/mL of final concentrations for PGE and 0.04–20 μg/mL for the pure compounds) were prepared in DMSO, then added to an assay mixture containing bovine serum albumin (150 mM), phosphate buffer (50 mM; pH 7.4), sodium azide (0.02% *w*/*v*), fructose (0.4 M), and glucose (0.4 M). The mixtures were incubated at 37 °C for 7 days, thereafter fluorescence was measured at an excitation wavelength of 355 nm and an emission of 460 nm. The inhibitory activity was calculated as percentage of the control, by using the following formula: (A_control_-A_sample_/A_control_) 100, where A_control_ is the fluorescence of the control, whereas A_sample_ is the fluorescence of the sample.

### 3.7. Cytoprotective Activity Under Hyperglycemia

#### 3.7.1. Cell Culture

The human gastric carcinoma (AGS) cells, kindly given by Prof. Gabriella Mincione (Department of Medical, Oral, and Biotechnological Sciences, University “G. d’Annunzio” of Chieti-Pescara, Italy), were grown at 37 °C in 5% CO_2_ in Dulbecco’s modified Eagle’s medium, supplemented with fetal bovine serum (10% *v*/*v*), glutamine (2 mM), streptomycin (100 µg/mL), and penicillin (100 U/mL). All experiments were performed when cells reached the logarithmic growth phase.

#### 3.7.2. Cytotoxicity Assay

The cells were seeded into 96-well microplates (2 × 10^4^ cells/well) and allowed to grow for 24 h; then, progressive dilutions of the PGE in ethanol (EtOH; 100% *v*/*v*) were added to cells (1% *v*/*v* in the cell medium). The vehicle was nontoxic at final concentration of 1% *v*/*v* in the medium. Cytotoxicity was measured up to 72 h incubation with the samples by the 3-[4,5-dimethylthiazol- 2-yl]-2,5-diphenyl tetrazolium bromide (MTT) assay according to previous published methods [84]. The assay was carried out three times and, in each experiment, each concentration was tested at least in six technical replicates. Cell viability was determined as percentage of the vehicle control.

#### 3.7.3. Cytoprotection Towards Oxidative Stress Induced by Hyperglycemia

For assessing the cytoprotective activity of PGE towards oxidative damages under hyperglycemia conditions, 5 × 10^5^ cells were plated into 6-well culture wells, allowed to grow for 24 h, then confluent cells (about 60–70% of confluence) were further exposed to hyperglycemic conditions (glucose concentration of 6.0 mg/mL) and progressive concentrations of PGE (10, 50, and 100 µg/mL) for 72 h. At the end of incubation, the intracellular levels of reactive oxygen species (ROS) and glutathione, as oxidative stress parameters, were measured as follows.

#### 3.7.4. Determination of Intracellular Levels of Reactive Oxygen Species (ROS)

The ROS levels induced by treatments were measured by the 2,7-dichlorofluorescein diacetate assay (DCFH-DA), according to Di Giacomo et al. [85] with slight changes. The DCF fluorescence, proportionally induced by ROS-mediated oxidative stress, was measured at an excitation wavelength of 485 nm and emission wavelength of 528 nm by a BD Accuri™ C6 flow cytometer. In each experiment, a vehicle control (corresponding to the basal ROS level) and a positive control (corresponding to the highest oxidation under hyperglycemic conditions) were included too. The mean DCF fluorescence of 10,000 cells was measured from all the treatments. The oxidation index was obtained by the ratio between the DCF fluorescence of sample and vehicle control.

#### 3.7.5. Chromatographic Determination of Intracellular Glutathione Levels

Intracellular levels of reduced (GSH) and oxidized (GSSG) glutathione were measured by HPLC-UV, according to previous standardized methods [86]. Briefly, cell pellets (1 × 10^6^) were suspended in 10% ice-cold TCA and centrifuged for 15 min at 9,000 *g*. The supernatant was collected, and GSH and GSSG were measured by HPLC with UV detection at 215 nm. The separation was achieved using an InfinityLab poroshell 120 EC-C18 column (3 × 150 mm, 2.7 μm) at a flow rate of 0.8 mL/min with the following elution gradient: 0–3 min 100% A + 0% B, 3–10 min from 100% A to 100% B. The composition of mobile phase A was 0.1% trifluoroacetic acid in water and mobile phase B was 0.1% trifluoroacetic acid in water/acetonitrile (93:7). In these chromatographic conditions, retention times were 2.58 min and 7.01 min for GSH and GSSG, respectively.

### 3.8. Statistical Analysis

All values are expressed as mean ± SEM or mean ± SD, as indicated. Statistical analysis was performed by GraphPad Prism™ (Version 4.00) software (GraphPad Software, Inc., San Diego, California, USA). The one-way analysis of variance (one-way ANOVA), followed by Dunnett’s Multiple Comparison Post Test, were used to analyze the difference between treatments. Moreover, unpaired data were analyzed with Student’s *t*-test. The concentration–response curves were constructed using the “Hill equation”: E = Emax/ [1 + (10LogEC50/A)HillSlope], where E is the effect at a given concentration of agonist, Emax is the maximum activity, IC_50_ is the concentration that produces a 50% of the inhibitory response, A is the agonist concentration in molarity, and HillSlope is the slope of the agonist curve. *p*-values of less than 0.05 (*p* < 0.05) were considered statistically significant.

## 4. Conclusions

The healing effects associated with pomegranate consumption, ascribed to the pleiotropic effects of its secondary metabolites, mainly phenolics, have been widely reported. Also, nonedible pomegranate peel has been highlighted to be enriched in phenolic acid and flavonoids with respect to edible parts, thus suggesting it could represent an environmentally-friendly nutraceutical source.

Here, we reported the hypoglycemic, antiglycation, and antioxidant cytoprotective properties of PGE, an ethyl acetate extract obtained by Soxhlet from the waste peel of the Dente di Cavallo DC2 Italian pomegranate variety. The extract was characterized by a 1:1 ratio between ellagic acid and rutin, which were the most abundant phenolics (about 4% *w*/*w*), along with lower amounts of catechin, gallic acid, 2,3-dimethoxy benzoic acid, syringic acid, and punicalagins.

This composition seems to be correlated with the ability of PGE to inhibit glycolytic enzymes and to affect AGE formation, likely through chelating mechanisms. Indeed, metal chelation has been highlighted to represent a primary mechanism by which inhibitors and breakers of AGE can stop their metal-catalyzed formation. In this context, the iron chelating activity of PGE can contribute to block AGE formation and to counteract the oxidative stress often associated to hyperglycemia. Accordingly, the literature data support the possible role of rutin, catechin, and gallic and ellagic acids as both AGE inhibitors and metal chelating agents. Moreover, these compounds were reported to inhibit α-amylase and α-glucosidase with different potency, likely due to their specific molecular structures.

Among the identified phenolics, ellagic acid and rutin were also found to be involved in the antioxidant cytoprotective effects of PGE in AGS cells, with ellagic acid being effective in reducing ROS levels, whereas rutin affected both ROS and GSH amounts. However, considering that synergistic and/or antagonistic interactions can occur within a phytocomplex, the true pharmacodynamic and/or pharmacokinetic contribution of all the different PGE phytochemicals remain to be clarified.

It is noteworthy that, although enhancing GSH capacity is an important aim to protect normal cells from redox-related changes or environmental toxins, depleting GSH levels may increase susceptibility of cancer cells to oxidative stress, thus representing a possible strategy for sensitizing cancer cells to chemotherapy. Further studies about the possible chemosensitizing role of PGE in gastric or other cancer cells are encouraged.

Altogether, these results provide preliminary scientific evidence about the ability of PGE to counteract oxidative and glycative stress associated with hyperglycemia by pleiotropic mechanisms, thus suggesting further studies to better characterize its possible usefulness as an antihyperglycemic remedy. Also, our results strengthen the interest for pomegranate peel as a natural source of bioactive phytochemicals or phytocomplexes, thus allowing it could be recycled as a starting material for further pharmaceutical and nutraceutical purposes.

## Figures and Tables

**Table 1:** Amounts of total phenolics, tannins, and flavonoids in the ethyl acetate extract by Soxhlet of the waste peel of *Punica granatum* L. var. Dente di Cavallo DC2 (PGE) (*n* = 6).

Compound	PGE µg/mg Sample (mean ± SD)
Phenolics (TAE) ^1^	206.3 ± 0.10
Tannins (TAE) ^1^	52.9 ± 0.06
Flavonoids (QE) ^2^	205.6 ± 14.70

^1^ TAE, tannic acid equivalents. ^2^ QE, quercetin equivalents.

**Table 2:** HPLC-PDA analysis of phenolics identified in the ethyl acetate extract, obtained by Soxhlet from the waste peel of *Punica granatum* L. var. Dente di Cavallo DC2 (PGE) (*n* = 3).

Compound	PGE μg/mg of Sample (Mean ± SD)
2,3-Dimethoxy benzoic acid	1.84 ± 0.11
Catechin	2.65 ± 0.11
Gallic acid	1.08 ± 0.09
Rutin	41.20 ± 3.80
Syringic acid	0.60 ± 0.06

**Table 3:** IC_50_ values of the ethyl acetate extract, obtained by Soxhlet from the waste peel of *Punica granatum* L. var. Dente di Cavallo DC2 (PGE), and the positive controls for enzyme inhibition, iron chelating activity, and inhibition of advanced glycation end-products (AGE).

Biological Activity Assay	PGE	Positive Controls
	IC_50_ (CL) μg/mL	
α-amylase inhibition	30.3 (13.4–67.3)	76.3 (46.2–126.0) ^1^
α-glucosidase inhibition	6.2 (5.8–6.7)	6.0 (2.7–9.4) ^1^ 7.3 (4.2–11.8)^2^
Lipase inhibition	nd	6.6 (1.4–12.3)^3^
Ferrous ion chelating activity	18.2 (17.4–19.1)	360.5 (299.9–486.8) ^2^
Ferric ion chelating activity	22.3 (7.6–69.1)	68.5 (54.9–85.7) ^4^
Inhibition of advanced glycation end-products (AGE)	142.3 (108.5–186.7)	41.5 (35.9–48.0) ^2^ 86.0 (77.3–97.5) ^5^

^1^ Acarbose; ^2^ rutin; ^3^ orlistat; ^4^ quercetin; ^5^ naringenin; nd, not determinable as the inhibition was lower than the 40%.

**Table 4:** Pearson correlation coefficient among biological activities of PGE.

	Pearson r (CL; R Square)				
	α-Amylase Inhibition	α-Glucosidase Inhibition	Fe^+2^ Chelating Activity	Fe^+3^ Chelating Activity	Age Inhibition
α-amylase inhibition	1	-	-	-	-
α-glucosidase inhibition	nsc	1	-	-	-
Ferrous ion chelating activity	0.99* (0.4–0.99; 0.99)	nsc	1	-	-
Ferric ion chelating activity	0.99* (0.6–0.99; 0.99)	nsc	0.99** (0.9–0.99; 0.99)	1	-
Inhibition of advanced glycation end-products (AGE)	nsc	nsc	0.91* (0.15–0.99; 0.83)	0.93* (0.28–0.99; 0.87)	1

nsc, not significantly correlated; CL, confidential limits. * *p* < 0.05 or ** and *p* < 0.01, statistically significant correlation (two-tailed *t*-test).

## Supplementary Materials

The following are available online, Figure S1: High-performance thin-layer chromatography (HPTLC) analysis of the peel extract from *Punica granatum* L. var. “Dente di cavallo” (PGE). Figure S2: Chromatograms of the peel extract from *Punica granatum* L. var. “Dente di cavallo” (PGE) obtained by high-performance liquid chromatography with photodiode array detection (HPLC-PDA; 278 nm).
