# Supplementary material for: Hypoglycemic, Antiglycation, and Cytoprotective Properties of a Phenol-Rich Extract From Waste Peel of Punica granatum L. var. Dente di Cavallo DC2
Source: Molecules. 2019 Aug 27;24(17):3103. doi: 10.3390/molecules24173103 (PMC6749322; doi:10.3390/molecules24173103)

*Supplementary materials*

*Article*

# Hypoglycemic, Antiglycation, and Cytoprotective Properties of a Phenol-Rich Extract From Waste Peel of *Punica granatum* L. var. Dente di Cavallo DC2

**Antonella Di Sotto [1,*], Marcello Locatelli [2,*], Alberto Macone [3], Chiara Toniolo [4], Stefania Cesa [5], Simone Carradori [2], Margherita Eufemi [3], Gabriela Mazzanti [1] and Silvia Di Giacomo [1]**

[1] Department of Physiology and Pharmacology "V. Erspamer", Sapienza University, P.le Aldo Moro 5, 00185 Rome, Italy

[2] Department of Pharmacy, University "G. D'Annunzio" of Chieti-Pescara, Via dei Vestini 31, 66100, Chieti, Italy

[3] Department of Biochemical Sciences "A. Rossi Fanelli", Sapienza University, P.le A. Moro 5, 00185 Rome, Italy

[4] Department of Environmental Biology, Sapienza University, P.le Aldo Moro 5, 00185 Rome, Italy

[5] Department of Chemistry and Technology of Drugs, Sapienza University, P.le Aldo Moro 5, 00185 Rome, Italy

* Correspondence: antonella.disotto@uniroma1.it (A.D.S.); m.locatelli@unich.it (M.L.)

**Figure S1.** High-performance thin-layer chromatography (HPTLC) analysis of the peel extract from *Punica granatum* L. var. "Dente di cavallo" (PGE). HPTLC silica gel 60 glass-backed layers were developed using the solvent mixture ethyl acetate:dichloromethane:acetic acid:formic acid:water (100:25:10:10:11 v/v/v/v/v) and derivatized by Natural Product Reagent (NPR) and/or anisaldehyde. (a) Visualization at 366 nm without derivatization. (b) Visualization at 366 nm after Natural Product Reagent (NPR) derivatization. (c) Visualization at 366 nm after anisaldehyde/NPR derivatization. (d) Visualization under white light after NPR and anisaldehyde derivatization. (e) Visualization at 254 nm. The chromatograms correspond to (1,2) *P. granatum* peel extract; (3) gallic acid; (4) rutin; (5) chlorogenic acid; (6) catechin; (7) caffeic acid; (8) quercetin; (9) kaempferol.

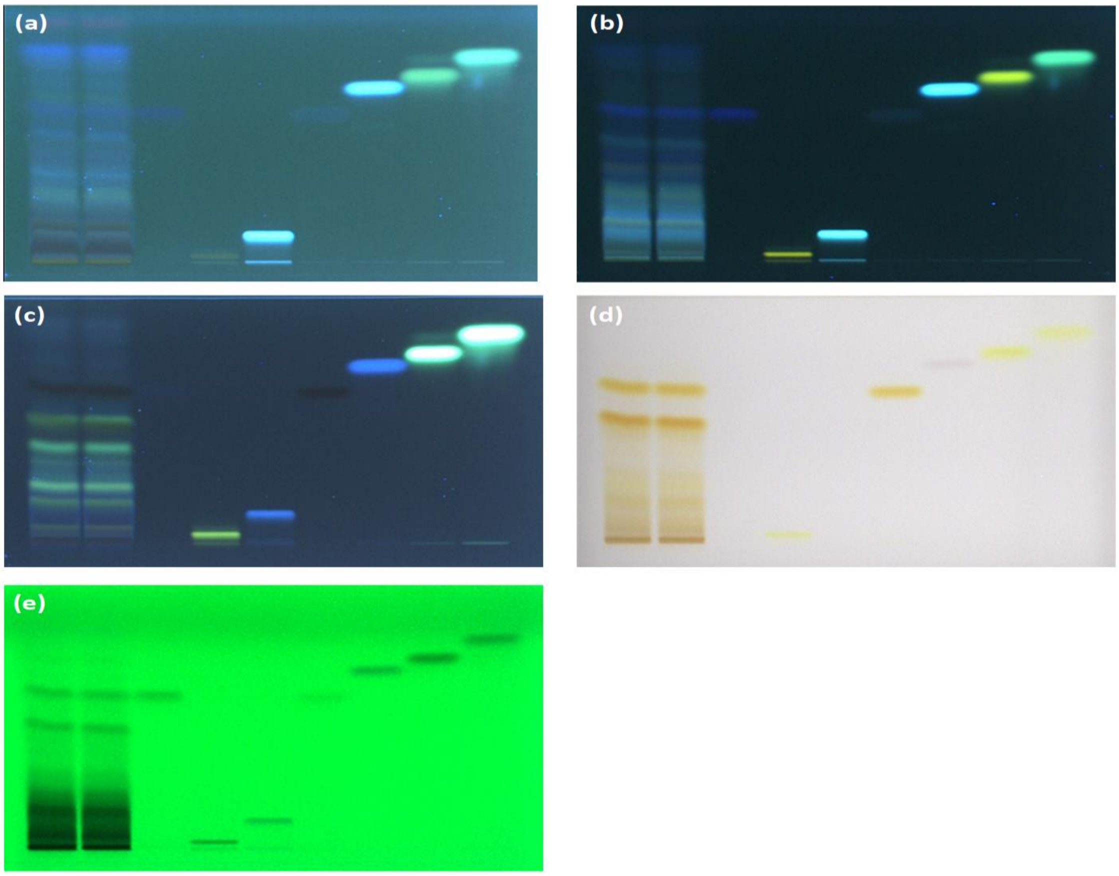

**Figure S2.** Chromatograms of the peel extract from *Punica granatum* L. var. “Dente di cavallo” (PGE) obtained by high-performance liquid chromatography with photodiode array detection (HPLC-PDA) (278 nm).

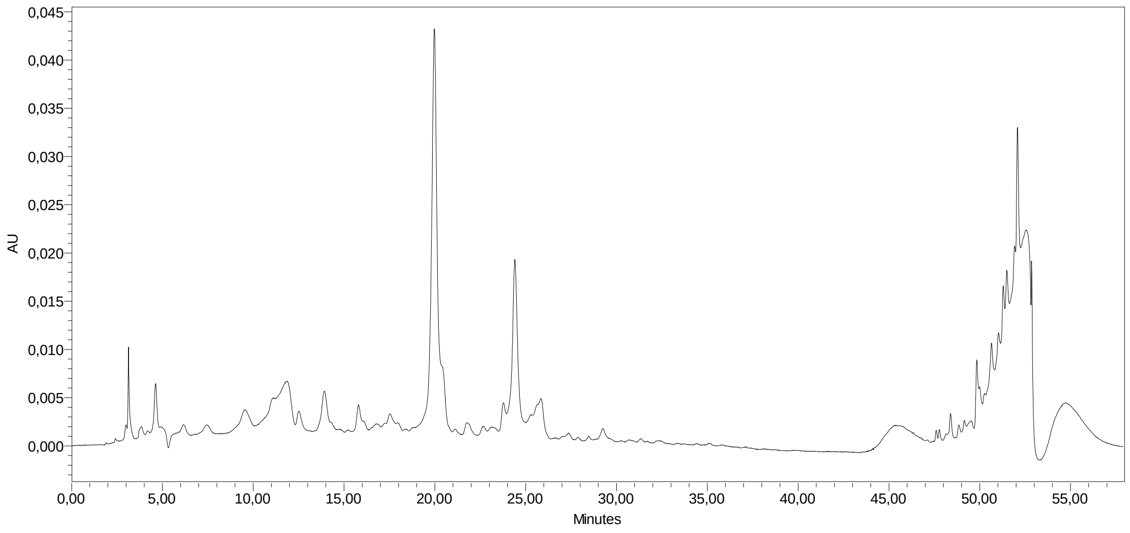

Supplement: Supplementary file 1 [file molecules-24-03103-s001.zip › molecules-575054-SI.pdf]
